# Possible Selves Across the Lifespan

**DOI:** 10.1093/geroni/igaa057.1454

**Published:** 2020-12-16

**Authors:** Victoria Chen, Alysson Light

**Affiliations:** University of the Sciences, Philadelphia, Pennsylvania, United States

## Abstract

Possible selves are defined as “personalized representations of one’s self in future states” (Cross & Markus, 1991). Research has also found that thinking frequently about possible selves predicts lower well-being, whereas thinking clearly about possible selves is associated with higher well-being (McElwee & Haugh, 2010). However, possible selves differ across the lifespan (Hooker & Kaus, 1994; Cross & Markus, 1991) and life stages can impact exploration of possible identities (Arnett, 2000). Thus we hypothesize that the relationship between thought about possible selves and well-being differs across the lifespan. In a cross-sectional survey, 240 participants (age range: 18-92) reported on frequency and clarity of possible selves, as well as general self-clarity (Campbell et al., 1996). Results indicate curvilinear moderation by age of the link between possible self-clarity and well-being, with the association being stronger among midlife adults than among younger adults and older adults. Also, as clarity of feared possible selves increases, self-concept clarity decreases. Similarly, frequency of thinking about feared possible selves was negatively correlated with self-concept clarity. However, clarity and frequency of thought about hoped-for positive possible selves had no correlation with self-concept clarity.

